# Association between three common genetic polymorphisms of *ATM* and the risk of schizophrenia

**DOI:** 10.17179/excli2021-4141

**Published:** 2021-08-25

**Authors:** Elham Abbasi, Mostafa Saadat

**Affiliations:** 1 Department of Biology, College of Sciences, Shiraz University, Shiraz 71467-13565, Iran

## ⁯⁯⁯⁯⁯⁯

***Dear Editor,***

Schizophrenia is a multifactorial disease and its pathogenesis is influenced by a broad range of genetic and environmental factors. Many genetic polymorphisms are associated with the risk of schizophrenia (Saadat, 2013[11]). There is a body of evidence indicating that increased oxidative stress and impaired DNA repair capacity are associated with increased risk of schizophrenia (Topak et al., 2018[15]). It has been well established that cellular DNA is regularly damaged by numerous endogenous and exogenous factors. Considering that unrepaired damage has serious consequences, damaged DNA should be recognized by specific DNA repair pathways. Common genetic variations in genes of DNA repair pathways have been identified which may alter the efficiency of DNA repair pathways (Hu et al., 2001[4]; Matullo et al., 2001[5]) and thereby contribute to the development of numerous diseases, including schizophrenia. Therefore, it has been suggested that the DNA repair pathway genes, such as *XPC*, *XPD*, *XRCC1*, and *XRCC3* might be associated with schizophrenia risk (Saadat et al., 2008[12]; Derakhshandeh et al., 2009[3]; Mazaheri and Saadat 2015[6]; Odemis et al., 2016[7]; Taghipour et al., 2019[14]). It has been reported that rare genetic variants in DNA repair genes are associated with aberrant hippocampal resting-state functional connectivity in schizophrenia (Yang et al., 2017[18]).

The *ATM* gene (MIM 607585) is one of the most significant genes in the DNA repair pathway encoding a vital checkpoint kinase. The ATM is critical in identifying and responding to DNA damage resulting in genome stability (Pizzamiglio et al., 2020[8]). Mutations in this gene cause ataxia-telangiectasia (AT), known as DNA instability syndrome, characterized by neural symptoms and cerebellar degeneration (Rothblum-Oviatt et al., 2016[10]). Data on the expression levels of eight putative biomarker genes including *ATM* have been used to discriminate between schizophrenia and healthy subjects (Tsuang et al., 2005[16]). Statistical analysis of combinations of 8 putative biomarker genes including the *ATM* gene expression levels is able to discriminate schizophrenia and healthy controls (Tsuang et al., 2005[16]). Thus, *ATM* is potentially a candidate gene involved in the development of schizophrenia. An association study showed no significant relationship between three common polymorphisms of *ATM* (rs600931, rs227061, and rs664143) and risk of schizophrenia (Zhang et al., 2008[19]). To the best of our knowledge, there is no study on the association between other *ATM* genetic polymorphisms (such as rs611646, rs609429, and rs228593) and risk of schizophrenia, therefore, the present case-control study was carried out.

In our previous report (Taghipour et al., 2019[14]) we provided a comprehensive description of the study participants. A total of 721 participants (361 schizophrenia patients and 360 healthy controls) were included in the study. Patients and controls were age- and sex-matched. Considering the heterogeneity of the Iranian population (Rafiee et al., 2010[9]), only the samples from Muslims living in Fars Province have been utilized. It is noteworthy that this study has been approved by Shiraz University's ethics committee. 

Here we used pooled samples for estimating the allelic frequency of the study polymorphisms in schizophrenia and control groups, as described previously (Saadat et al., 2019[13]). The PCR-RFLP based method was used using primers specific to rs611646, rs609429 (IVS48+238 C>G), and rs228593 polymorphisms (Table 1(Tab. 1)). The primer for rs611646 was designed with Allele ID software version 7.50, and the other two primers were based on previous researches (Akulevich et al., 2009[1]; Wang et al., 2010[17]). The OR (odds ratio) and the 95 % confidence interval (95 % CI) indicate the association between the polymorphisms with the disease. The data were analyzed by SPSS software version 25 with a significance level of less than 0.05. 

There is a significant difference between control and patient groups for the allelic frequencies of the rs609429 polymorphism (OR=1.33, 95 % CI=1.05-1.68, *P*=0.020). The frequency of the G allele of rs609429 is positively associated with the risk of schizophrenia. Statistical analysis revealed no significant association between the allelic frequencies of rs611646 and rs228593 polymorphisms and the risk of schizophrenia (Table 2(Tab. 2)).

It should be noted that the rs609429 is an intronic genetic variation (IVS48+238, C>G) and seems to be involved in splicing, therefore, it is a functional polymorphism. The G allele leads to generation of a weak additional splicing region and subsequently, reduces the expression of ATM gene (Angèle et al., 2003[2]). It might be concluded that individuals carrying the G (variant) allele have lower expression level and DNA repair capacity and are more susceptible to schizophrenia compared to whom carrying the C (ancestral) allele.

Our present data suggest that the *ATM* gene is a candidate locus for susceptibility to schizophrenia and the variant G allele of the rs609429 polymorphism may provide valuable insight into its association with schizophrenia. More association studies on larger samples from other populations are necessary to reveal the relevance of *ATM* polymorphisms as potential risk factors for schizophrenia and provide a deeper understanding of the genetic causes of the disease.

## Acknowledgements

The authors are indebted to the participants for their close cooperation. 

## Conflict of interest

None. 

## Figures and Tables

**Table 1:**
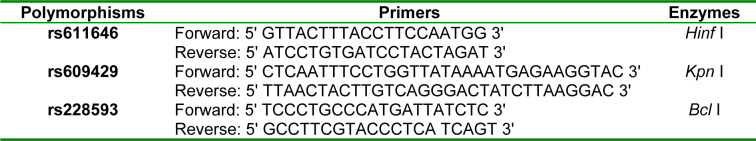
Primers and restriction enzymes used in PCR-RFLP

**Table 2:**
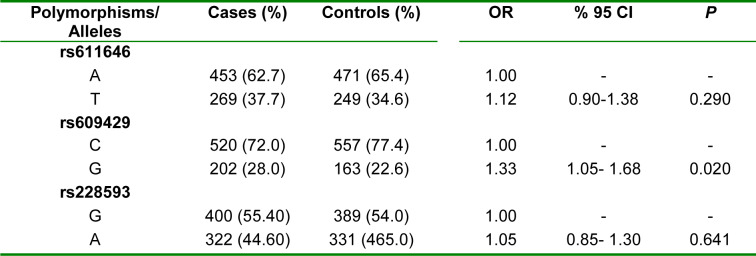
Allelic frequency of the *ATM* rs611646, rs609429, and rs228593 polymorphisms in controls and schizophrenia patients
